# Methodological concerns regarding stepwise regression and construct overlap in caregiver burden research

**DOI:** 10.1590/1980-5764-DN-2026-0475

**Published:** 2026-04-03

**Authors:** Daniel Souza Gonçalves de Araujo

**Affiliations:** 1 Universidade de São Paulo, Faculdade de Medicina – São Paulo SP, Brazil.

Dear Editor,

I read with great interest the article "Factors associated with family caregiver burden of patients with dementia" by Hermes-Pereira et al.^1^, recently published in *Dementia & Neuropsychologia*. The study addressed a topic of high clinical relevance by identifying depressive symptoms, caregiver distress, and quality of life as predictors of burden. However, I write to express significant concerns regarding the statistical methodology employed, specifically the use of stepwise regression preceded by univariate pre-screening, and the interpretation of predictors that likely suffer from substantial construct overlap.

My primary concern involves the use of automated stepwise variable selection. While this technique remains common in medical literature, it has been widely criticized by statisticians for decades. As demonstrated by Babyak^2^ and Whittingham et al.^3^, automated algorithms capitalize on random sampling error to select predictors. This process frequently results in overfitting, yielding models that fit the specific sample data exceptionally well but fail to replicate in independent samples. The high adjusted R^2^ of 0.52 reported in the final model is likely an optimistic estimate driven by this algorithmic data dredging rather than a true reflection of population parameters^2,4^.

In addition, the study's reliance on univariate Spearman correlations to pre-screen variables, such as excluding anxious symptoms and patient functionality due to p>0.05, introduces the problem of phantom degrees of freedom^5^. Degrees of freedom are expended during this screening process, yet this statistical cost is ignored in the final model calculations. Consequently, the standard errors are underestimated, and the reported p-values are invalid and misleadingly small^2,5^. This effectively inflates the risk of Type I errors and presents exploratory findings with an unjustified level of certainty.

I must also address the issue of collinearity and construct redundancy regarding the finding that depressive symptoms, as measured by the BDI-II, predicted 34% of caregiver burden, as measured by the ZBI, in the first model. From a psychometric perspective, this high correlation is expected and potentially tautological. An analysis of the instruments reveals significant semantic overlap. Items in the ZBI assessing exhaustion, irritability, and loss of social life are phenomenologically indistinct from items in the BDI-II assessing fatigue, irritability, and social withdrawal^6,7^. When a predictor such as depression and an outcome such as burden measure the same underlying latent constructs, the regression model does not identify a causal predictor but rather correlates the symptom with itself. Harrell^8^ warns that in the presence of such collinearity, stepwise algorithms are highly unstable and arbitrarily select one variable over another based on minor fluctuations in the data. The dominance of depression in this model may simply reflect this structural redundancy rather than a distinct clinical etiology.

The stepwise procedure also induces a systematic bias in parameter estimation. Steyerberg et al.^9^ and Derksen and Keselman^10^ have shown that coefficients selected via stepwise methods are biased away from zero. The reported coefficient for depressive symptoms is likely exaggerated, which may lead clinicians to overestimate the potential impact of treating depression by reducing objective burden.

To ensure the robustness of future research in this field, I suggest prioritizing full model specifications, with predictors selected a priori based on clinical theory^2,8^, over stepwise procedures. Additionally, researchers should account for the overlap between psychometric scales to avoid interpreting measurement redundancy as predictive power.

## Data Availability

The datasets generated and/or analyzed during the current study are available from the corresponding author upon reasonable request.
